# Structure and Change: New Developments in Research on Cumulative Dis/Advantage

**DOI:** 10.1093/geroni/igab046.1955

**Published:** 2021-12-17

**Authors:** William Dannefer, Carroll Estes

## Abstract

In a time of heightened social inequality and concern to reckon with its sources and consequences, the relevance of cumulative dis/advantage (CDA) to understanding patterns of aging has become even clearer, and CDA research has continued to expand in several fresh directions. Papers in this symposium will review the current state of knowledge regarding CDA and will present new analyses addressing key questions of its intersections with social change and its structural patterning. We will begin with a review of knowledge on comparative evidence regarding cumulative dis/advantage and its cross-national patterning. With regard to change, will examine the compare the effect of the 2008 recession and subsequent recovery across generational cohorts through a comparative examination of trajectories of income inequality. We will also present evidence on the impact of gender, focusing on women’s late-life health.

